# Impact of personal goals on the internal medicine R4 subspecialty match: a Q methodology study

**DOI:** 10.1186/1472-6920-13-171

**Published:** 2013-12-21

**Authors:** Vijay J Daniels, Narmin Kassam

**Affiliations:** 1 Department of Medicine, University of Alberta, Edmonton, Canada

**Keywords:** Career choice, Internal medicine, Q methodology, Postgraduate medical education, Specialization, Canada

## Abstract

**Background:**

There has been a decline in interest in general internal medicine that has resulted in a discrepancy between internal medicine residents’ choice in the R4 subspecialty match and societal need. Few studies have focused on the relative importance of personal goals and their impact on residents’ choice. The purpose of this study was to assess if internal medicine residents can be grouped based on their personal goals and how each group prioritizes these goals compared to each other. A secondary objective was to explore whether we could predict a resident’s desired subspecialty choice based on their constellation of personal goals.

**Methods:**

We used Q methodology to examine how postgraduate year 1–3 internal medicine residents could be grouped based on their rankings of 36 statements (derived from our previous qualitative study). Using each groups’ defining and distinguishing statements, we predicted their subspecialties of interest. We also collected the residents’ first choice in the subspecialty match and used a kappa test to compare our predicted subspecialty group to the residents’ self-reported first choice.

**Results:**

Fifty-nine internal medicine residents at the University of Alberta participated between 2009 and 2010 with 46 Q sorts suitable for analysis. The residents loaded onto four factors (groups) based on how they ranked statements. Our prediction of each groups’ desired subspecialties with their defining and/or distinguishing statements are as follows: group 1 – general internal medicine (variety in practice); group 2 – gastroenterology, nephrology, and respirology (higher income); group 3 – cardiology and critical care (procedural, willing to entertain longer training); group 4 – rest of subspecialties (non-procedural, focused practice, and valuing more time for personal life). There was moderate agreement (kappa = 0.57) between our predicted desired subspecialty group and residents’ self-reported first choice (p < 0.001).

**Conclusion:**

This study suggests that most residents fall into four groups based on a constellation of personal goals when choosing an internal medicine subspecialty. The key goals that define and/or distinguish between these groups are breadth of practice, lifestyle, desire to do procedures, length of training, and future income potential. Using these groups, we were able to predict residents’ first subspecialty group with moderate success.

## Background

There is a discrepancy between societal need for general internal medicine specialists and internal medicine residents’ choice in the R4 subspecialty match [1-7]. There has been a decline in internal medicine residents’ interest in general internal medicine in the United States with 54% planning to practice general internal medicine in 1998 [8] compared with 22% from survey data collected from 2008 to 2011 [9]. There are similar concerns in South Asia [4] and in several European countries [5-7]. In Canada, internal medicine residency training begins with three years of core internal medicine, and then all residents apply and match (known as the R4 match) to a subspecialty residency with general internal medicine as one of those options. Between 2008 and 2010, 21-24% of internal medicine residents pursued general internal medicine based on Canadian Post M.D. Education Registry (CAPER) data [10]. Concurrently, Canadian physician resource studies predicted 400–500 general internists would retire between 2010 and 2013 with the number of new graduates only meeting half of this demand, let alone the current deficit in many areas of the country and the expected population growth [11].

There have been several studies published examining demographic and non-demographic aspects associated with internal medicine residents’ choice of subspecialty [8,12-21]. However, few have looked at the relative importance of personal goals and their impact on the internal medicine R4 subspecialty match [8,14,16]. These studies examined personal goals based on career path with participants being surveyed with either dichotomous (yes or no) items [8] or five-point likert scales [14,16]. Although these approaches can answer the question, “Does this group value lifestyle?”, they may not be the ideal methods to answer the question “How high of a priority is lifestyle for this group as compared to others?” as there is nothing that forces respondents to prioritize the items they answer.

The purpose of this study was to assess if internal medicine residents can be grouped based on their personal goals and how each group prioritizes these goals compared to each other. A secondary objective was to explore whether we could predict a resident’s desired subspecialty choice based on their constellation of personal goals.

## Methods

### Study setting

This study was conducted at the University of Alberta with postgraduate year (PGY) one to three internal medicine residents.

### Design

The design framework was Q methodology invented in 1935 by British physicist-psychologist William Stephenson to allow quantitative analysis of subjective matters [22]. Cross [23] describes Q methodology as the ideal method to measure attitudes as it involves a forced distribution greatly limiting the number of uncertain responses as compared to a likert scale. Q methodology involves the sorting of statements (the Q set) into columns of Agree to Disagree and then Q sets are analyzed with a factor analysis. However unlike traditional factor analysis where items are loaded onto a factor, in Q methodology, the respondents are loaded onto a factor. This results in factors or groups of respondents who ranked statements similarly. The output of these analyses is accompanied by standard scores (z-scores) for each statement for a given factor.

### Q set

**Figure 1:**
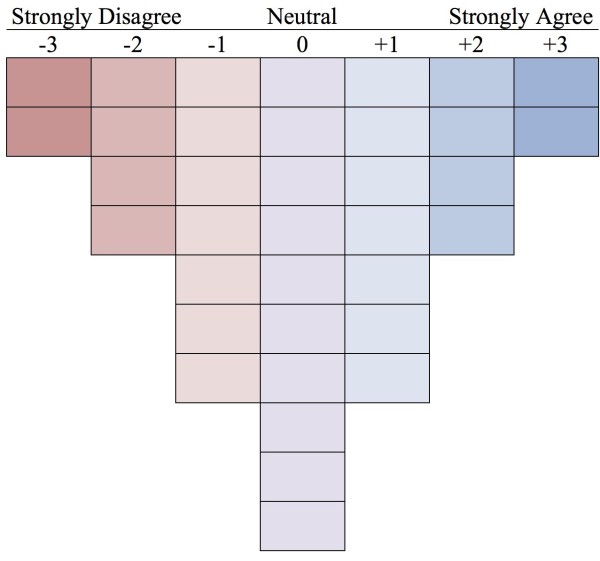
Q sort sheet.

We began with all of the quotes from our previous qualitative study [19], and each author independently created a set of representative statements. The authors then discussed the statements and created a list of 36 representative statements (see Table 1). These statements were then placed into a score sheet with a quasi-normal distribution (see Figure 1).

**Table 1:** Q set (statements included in the Q sort)

**Statement**	
1.	My decision is based on the physiology of the subspecialty.
2.	GIM doesn’t get any of the interesting single organ diseases.
3.	The patients in my subspecialty of interest are fascinating.
4.	I really like the general medicine population of patients.
5.	I prefer to be an expert in my area.
6.	With the amount of knowledge, you cannot keep up with GIM.
7.	GIM patients are too complex.
8.	The positive interactions I have had with attendings affected my decision.
9.	The negative interactions I have had with attendings affected my decision.
10.	My decision is predominantly based on experiences as a medical student.
11.	My decision is predominantly based on experiences as a resident.
12.	I value more time for my personal life.
13.	Lifestyle and more control over my practice are important.
14.	I want to be in a field that is in demand.
15.	I am focused on my job prospects.
16.	I want to be in a large city.
17.	I want to work in a smaller community.
18.	The resources of a fellowship program are important.
19.	The skills I need are easier to attain from a subspecialty fellowship than a GIM fellowship.
20.	I want something that pays well given how long I’ve trained.
21.	I like a little bit of everything and variety.
22.	Some specialties are not fairly remunerated & this is an important consideration.
23.	I prefer an outpatient-based practice.
24.	I prefer an inpatient-based practice.
25.	I'd like to do a combination of inpatient and outpatient.
26.	I enjoy procedures.
27.	I want to do procedures as part of my practice.
28.	I want to be done after five years of residency.
29.	I am willing to do as many years as it takes to get the job and subspecialty I want.
30.	I like the fact you can save 1+ years in GIM.
31.	A two-year GIM fellowship is a deterrent.
32.	The fact that subspecialists can also be general internists is important to me.
33.	Prestige and respect from colleagues matter to me.
34.	Prestige and respect from the public matter to me.
35.	I want to be valued in what I’m doing.
36.	GIM is a dumping ground for patients nobody wants.

### Study participants and data collection procedures

Ethics approval for the study was granted by the Health Research Ethics Board – Panel B at the University of Alberta. With permission of the internal medicine residency program, we approached the PGY-1 to PGY-3 internal medicine residents at one of their Academic Half-Days at the start of the 2009 academic year to explain the project and ask for volunteers to participate in our study. Consenting participants were given randomly sorted cards, each with one of the 36 statements. They were first asked to rank the 36 statements into levels of agreement and disagreement. Each participant then entered the number corresponding to each statement onto the score sheet (see Figure 1). Each Q-sort was considered adequate for analysis if all statements were used exactly once.

Participants were also asked to indicate their first three desired subspecialties and enter demographic information such as age range, gender, year of residency, and highest level of education (Bachelor’s, Master’s, Ph.D.). The procedure was repeated at the start of the 2010 academic year to include residents who were not present at the 2009 session (those that missed the session and new residents to the program).

### Data analysis

As mentioned previously, Q Methodology uses a factor analysis, but instead of grouping items measuring a similar construct, it groups respondents who rank statements similarly. PQMethod 2.11 [24] was used to enter and analyze the Q sort data. We performed factor analysis by principal component analysis with subsequent varimax rotation and compared the rotations of three to six factors choosing the most adequate solution. Adequacy is determined individually for a study [25]; we felt the most adequate solution would: 1) balance between maximizing the respondents loading onto factors with the variance explained, and 2) be judged by three independent evaluators (V.J.D., N.K., D.B.R.) as appearing internally consistent. This latter decision was based on the sorted statements that are defining statements and distinguishing statements for that factor. Defining statements are those with z-scores greater than +1 (factor agreed with these statements) or less than -1 (factor disagreed with these statements), and distinguishing statements are those that a particular factor agreed or disagreed statistically more strongly than other factors [26].

Once we had identified the number of factors, the same three independent evaluators examined each factor’s defining and distinguishing statements to predict the desired subspecialties of the residents that loaded onto a factor. To make these predictions, two of the evaluators (D.B.R. and N.K.) used their experiences as residency program directors for core internal medicine and general internal medicine respectively in which they counselled many residents on their selection of residency program, and the third evaluator (V.J.D.) used recent experiences as a resident and discussions with colleagues during training. Each evaluator predicted subspecialties for each factor, and then discussed their thoughts with any disagreements resolved by consensus. We used SPSS v.21 [27] to calculate a kappa coefficient to analyze for agreement between our predicted subspecialty group and the residents’ self-reported first desired subspecialty group. We also performed Mann Whitney U tests to assess differences in age grouping, year of residency, and level of education, and chi-squared tests to assess for differences in gender and first subspecialty choice between participants who were included in the comparison of predicted and self-reported subspecialty group and those who were not.

## Results

**Figure 2:**
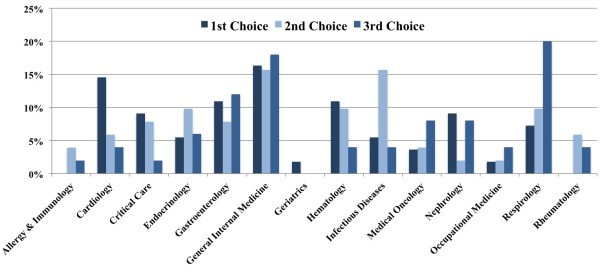
Desired choice of subspecialty.

In 2009, 43 residents participated with an additional 16 in 2010 (total 59) out of a possible 114 residents. Thirteen Q sorts were excluded because of either missing or duplicate statements resulting in 46 Q sorts suitable for analysis. Residents were primarily 26 to 31 years old (63% of participants), with 16% younger than 26 years old and 21% older than 31 years old. The majority (63%) were female, and 42% were in PGY-1, 28% in PGY-2 and 30% in PGY-3. Nine percent of participants did not have a premedical degree, 63% had a bachelor’s degree and 28% had a master’s degree. Participants desired choice of subspecialties is shown in Figure 2.

We compared the different solutions from rotating three to six factors in terms of number of Q sorts that loaded onto a factor and percentage of variance explained (see Table 2). We deemed four or five factors to have the adequate number of Q sorts and percentage of variance explained. The three evaluators (V.J.D., N.K. and D.B.R.) independently reviewed each solution based on each factor’s defining and distinguishing statements; each evaluator felt the four-factor solution was the most appropriate.

**Table 2:** Number of factors rotated with number of Q sorts loaded and variance explained

	**Number of factors rotated**			
	**3**	**4**	**5**	**6**
**# of Q sorts that loaded onto factors (total 46)**	29	35	34	27
**Variance explained**	48%	54%	59%	63%

The key statements that define and/or distinguish between these factors are breadth of practice, lifestyle, desire to do procedures, length of training, and future income potential. Three statements with the most agreement between factors are as follows: “I’d like to do a combination of inpatient and outpatient” (moderate agreement from the four factors); “My decision was predominantly based on experiences as a medical student” (mild disagreement from the four factors); and “Prestige and respect from colleagues matters to me” (scored as neutral by all four factors). The three statements with the most disagreement between factors were: “I am willing to do as many years as it takes to get the job and subspecialty I want” (moderate agreement by factor 3 but disagreement from all other factors); “I like a little bit of everything and variety” (strong agreement from factor 1, mild agreement or neutral from other factors); and “A two-year GIM fellowship is a deterrent” (mild agreement from factor 2 but disagreement from other factors).

The three evaluators reviewed the defining and distinguishing statements (see Tables 3, 4, 5 and 6) and predicted that factor 1 represents those interested in general internal medicine (variety as the top priority), factor 2 represents gastroenterology, nephrology, and respirology (higher income), factor 3 represents cardiology and critical care (longer training, less concern with time for personal life), and factor 4 represents the rest of the subspecialties (lower income, non-procedural subspecialties). Although 35 residents loaded onto a factor, one did not provide their desired subspecialty. For the remaining 34 residents there was moderate agreement (kappa = 0.57, p < 0.001) between our predictions of what each factor would choose as their subspecialty group and residents’ self-reported first desired subspecialty (see Table 7). There were no differences in age (p = 0.13), year of residency (p = 0.18), level of education (p = 0.66), gender (p = 0.72) or first subspecialty choice (p = 0.55) between participants who were included in the comparison of predicted and self-reported subspecialty group and those who were not.

**Table 3:** Defining and distinguishing statements for factor 1 (n = 8)

**No.**	**Statement**	**Rank**	**Z-score**
**Defining statements**			
Agrees strongly with the following statements			
21	I like a little bit of everything and variety	3	2.71
13	Lifestyle and more control over my practice are important	3	1.81
12	I value more time for my personal life	2	1.38
25	I’d like to do a combination of inpatient and outpatient	2	1.15
27	I want to do procedures as part of my practice	2	1.08
Disagrees strongly with the following statements			
24	I prefer an inpatient-based practice	-2	-1.07
9	The negative interactions with attendings were important	-2	-1.18
29	I am willing to do as many years as it takes to get the job and subspecialty I want	-2	-1.30
6	With the amount of knowledge, you can not keep up with GIM	-3	-1.31
2	GIM doesn’t get any of the interesting single organ diseases	-3	-1.99
**Distinguishing statements**			
Agrees with the following statements more than other factors			
21	I like a little bit of everything and variety	3	2.71*
4	I really like the general medicine population of patients	1	0.78
7	GIM patients are too complex	0	0.30*
17	I want to work in a smaller community	0	-0.17*
Disagrees with the following statements more than other factors			
35	I want to be valued in what I’m doing	0	0.48
5	I prefer to be an expert in my area	0	-0.22*
32	The fact that subspecialists can also be general internists is important to me	-1	-0.61*
16	I want to be in a large city	-1	-0.71*
24	I prefer an inpatient-based practice	-2	-1.07*
2	GIM doesn’t get any of the interesting single organ diseases	-3	-1.99

Factor 1 predicted to represent those interested in general internal medicine.

All distinguishing statements are p<0.05 and * indicates p<0.01.

**Table 4:** Defining and distinguishing statements for factor 2 (n = 9)

**No.**	**Statement**	**Rank**	**Z-score**
**Defining statements**			
Agrees strongly with the following statements			
12	I value more time for my personal life	3	1.59
16	I want to be in a large city	3	1.53
32	The fact that subspecialists can also be general internists is important to me	2	1.20
20	I want something that pays well given how long we’ve trained	2	1.13
25	I’d like to do a combination of inpatient and outpatient	2	1.12
13	Lifestyle and more control over my practice are important	2	1.10
31	A two-year GIM fellowship is a deterrent	1	1.04
35	I want to be valued in what I’m doing	1	1.02
14	I want to be in a field that is in demand	1	1.01
Disagrees strongly with the following statements			
36	GIM is a dumping ground for patients nobody wants	-2	-1.13
9	The negative interactions with attendings were important	-2	-1.21
7	GIM patients are too complex	-2	-1.33
23	I prefer an outpatient-based practice	-2	-1.33
29	I am willing to do as many years as it takes to get the job and subspecialty I want	-3	-1.94
17	I want to work in a smaller community	-3	-2.33
**Distinguishing statements**			
Agrees with the following statements more than other factors			
16	I want to be in a large city	3	1.53*
32	The fact that subspecialists can also be general internists is important to me.	2	1.20*
31	A two-year GIM fellowship is a deterrent	1	1.04*
22	Some specialties are not fairly remunerated and this is an important consideration	1	0.76*
Disagrees with the following statements more than other factors			
18	The resources of a fellowship program are important	-1	-0.51
11	My decision was predominantly based on experiences as a resident	-1	-0.90*
29	I am willing to do as many years as it takes to get the job and subspecialty I want	-3	-1.93*

Factor 2 predicted to represent those interested in gastroenterology, nephrology, & respirology.

All distinguishing statements are p<0.05 and * indicates p<0.01.

**Table 5:** Defining and distinguishing statements for factor 3 (n = 7)

**No.**	**Statement**	**Rank**	**Z-score**
**Defining statements**			
Agrees strongly with the following statements			
5	I prefer to be an expert in my area	3	1.50
35	I want to be valued in what I’m doing	3	1.44
29	I am willing to do as many years as it takes to get the job and subspecialty I want	2	1.38
1	My decision is based on the physiology of the subspecialty	2	1.37
36	GIM is a dumping ground for patients nobody wants	2	1.32
27	I want to do procedures as part of my practice	2	1.12
Disagrees strongly with the following statements			
28	I want to be done after five years of residency	-1	-1.13
2	GIM doesn’t get any of the interesting single organ diseases	-2	-1.25
23	I prefer an outpatient-based practice	-2	-1.30
6	With the amount of knowledge, you can not keep up with GIM	-2	-1.45
31	A two-year GIM fellowship is a deterrent	-2	-1.79
30	I like the fact you can save 1+ years in GIM	-3	-1.88
17	I want to work in a smaller community	-3	-1.89
**Distinguishing statements**			
Agrees with the following statements more than other factors			
29	I am willing to do as many years as it takes to get the job and subspecialty I want.	2	1.38*
1	My decision is based on the physiology of the subspecialty	2	1.37*
36	GIM is a dumping ground for patients nobody wants	2	1.32*
Disagrees with the following statements more than other factors			
12	I value more time for my personal life	0	-0.03*
28	I want to be done after five years of residency	-1	-1.13*
31	A two-year GIM fellowship is a deterrent	-2	-1.79*
30	I like the fact you can save 1+ years in GIM	-3	-1.88*

Factor 3 predicted to represent those interested in cardiology and critical care.

All distinguishing statements are p<0.05 and * indicates p<0.01.

**Table 6:** Defining and distinguishing statements for factor 4 (n = 11)

**No.**	**Statement**	**Rank**	**Z-score**
**Defining statements**			
Agrees strongly with the following statements			
5	I prefer to be an expert in my area	3	1.95
13	Lifestyle and more control over my practice are important	3	1.92
12	I value more time for my personal life	2	1.62
3	The patients in my subspecialty of interest are fascinating	2	1.45
35	I want to be valued in what I’m doing	2	1.41
8	The positive interactions with attendings were important	2	1.36
Disagrees strongly with the following statements			
22	Some specialties are not fairly remunerated and this is an important consideration	-2	-1.04
17	I want to work in a smaller community	-2	-1.05
27	I want to do procedures as part of my practice	-2	-1.33
2	GIM doesn’t get any of the interesting single organ diseases	-2	-1.47
7	GIM patients are too complex	-3	-1.53
26	I enjoy procedures	-3	-1.74
**Distinguishing statements**			
Agrees with the following statements more than other factors			
3	The patients in my subspecialty of interest are fascinating	2	1.45
8	The positive interactions with attendings were important	2	1.36*
28	I want to be done after five years of residency	1	0.58*
9	The negative interactions with attendings were important	0	0.23*
Disagrees with the following statements more than other factors			
20	I want something that pays well given how long we’ve trained	0	-0.63*
27	I want to do procedures as part of my practice	-2	-1.33*
26	I enjoy procedures	-3	-1.74*

Factor 4 predicted to represent those interested in non-procedural, lower income subspecialties.

All distinguishing statements are p<0.05 and * indicates p<0.01.

**Table 7:** Agreement of predicted subspecialty group and self-reported first desired subspecialty group

		**Self-reported first desired subspecialty group**				**Total**
		**1.00**	**2.00**	**3.00**	**4.00**	
Predicted	1.00	5	1	0	1	7
Subspecialty	2.00	0	5	3	1	9
Group	3.00	0	1	5	1	7
(Q sort Factor)	4.00	2	0	1	8	11
Total		7	7	9	11	34

Kappa = 0.57, p < 0.001.

## Discussion

The purpose of this study was to evaluate if we could group residents based on how they prioritized their personal goals for their career, and we were able to group 35 of 46 residents (76%) to one of four groups. Using these personal goals groups, we had moderate agreement (kappa = 0.57) between our predicted subspecialty group and residents’ self-reported first desired subspecialty, something that we have not seen in our review of the literature.

The main advantage of Q methodology is that it forces a quasi-normal distribution so respondents cannot select the same response for every item, as they can with traditional survey methods. Horn and colleagues’ [14] Canadian survey study grouped their respondents based on subspecialties (procedural, non-procedural, and non-procedural with declining interest) and found that all three groups valued diversity of clinical spectrum. We found that only one group valued variety compared to other groups. Similarly, Horn and colleagues [14] found their three groups all valued satisfaction among staff physicians whereas only one of our groups valued positive interactions with attending physicians. Although we are not comparing the exact same statements, the differences between these two studies are likely attributable to the methodology, with Q methodology allowing better differentiation between groups.

Examining the four groups of residents (see Tables 3, 4, 5 and 6), there are some key findings. It has been established in medical students that interest in a specialty correlates strongly with future income [28]. In internal medicine, a common finding across studies is that subspecialties such as cardiology, gastroenterology, hospitalists, nephrology, and respirology value a higher remuneration more than other subspecialties. We found that the group of residents interested in gastroenterology, nephrology and respirology valued remuneration more than others, but this was not the driver for those interested in cardiology or general internal medicine (which in Canada is somewhat similar to a hospitalist in the United States). The primary driver for those interested in cardiology and critical care was willingness to extend training, which is consistent with our anecdotal experience of these residents often doing seven, eight or sometimes more years of residency before ending training.

In our previous qualitative study [19], we often heard residents referring to general internal medicine as a dumping ground for patients other subspecialties did not want. In this study, it would appear that this sentiment is only a strong motivator for those interested in cardiology and critical care and was not a major concern for the other groups. In Canada, we have recently moved from a one to two-year general internal medicine fellowship after three core years of internal medicine. Our previous study indicated this increased length of training may be a deterrent to pursuing general internal medicine, and we have now found that this applies mainly to those interested in gastroenterology, nephrology and respirology.

In the current study, it was hard to tease out the non-procedural lower income subspecialties from each other, which may be due to a smaller sample size. However, this may also be due to a similar set of goals with these residents: they do not want to do procedures, are not as concerned with income, and want a focused area of expertise. What may distinguish them from each other is the specific patients whom they are interested seeing and the positive and negative interactions the residents have had with attending physicians.

Because this study was done with Canadian residents, it is worth noting the similarities and differences in the practice of general internal medicine in Canada compared to other countries. Ghali and colleagues [29] reviewed the clinical profile of general internal medicine in Argentina, Australia, Canada, Japan, New Zealand, Switzerland, and the United States. General internal medicine in Canada is a consultative service to primary care practitioners and is often hospital based caring for complex, multisystem patients. This overall profile is similar to that of general internists in Australia and New Zealand, and somewhat similar to general internists in Switzerland. This differs from general internists in the United States, Argentina and Japan who often have a large outpatient primary care role, though many also have consultative and hospitalist roles.

That said, one of the most interesting findings of our study is why a resident desires a career in general internal medicine. It is not surprising this group enjoys variety but what is surprising is this is essentially the sole driver for choosing general internal medicine over other subspecialties as exhibited by the extremely high Z-score of 2.71. A past president of the Canadian Society of Internal Medicine may have put it best when he said, “To my mind, this clinical smorgasbord is the best advertisement for doing general internal medicine” [30]. Regardless of the differences in the clinical profile of general internal medicine globally, this thirst for variety as a driver for residents to pursue general internal medicine is likely to transcend geographical borders.

### Limitations and future directions

This study was undertaken using residents at one internal medicine residency program in Canada and thus the results may be different at other institutions across Canada or in other countries. We were able to sample 59 (52%) of the 114 eligible residents; however, 13 did not complete the Q sort successfully. This resulted in only 46 participants of whom 11 did not load onto a factor and one did not complete their desired subspecialty, so they could not be included in our prediction of subspecialty compared to self-reported desired subspecialty. Though the demographics of the remaining 34 participants appear similar to the rest, this reduction in participants may reduce the generalizability of our findings. Finally, the statements used, though diverse, did not incorporate every possible personal goal such as desire for long versus short-term relationships with patients, debt load when entering residency, interest in specific issues such as health care policy, or desire for an academic versus non-academic career.

A future Q methodology study could involve residents across Canada, which might allow us to get at a more granular level (possibly 14 factors that could predict the individual subspecialties). Future research could also incorporate personal goals that were missing from this study, and could be done in a computerized format to prevent missing data. This would be helpful to further elucidate the impact of personal goals on residents’ choice in the R4 subspecialty match.

## Conclusion

Although previous aspects identified in the literature are important, this study suggests that residents fall into four groups based on a constellation of personal goals when choosing an internal medicine subspecialty. The key goals that define and/or distinguish between these groups are breadth of practice, lifestyle, desire to do procedures, length of training, and future income potential. Using these personal goals groups, we were able to predict residents’ first subspecialty choice/group with moderate success.

## Competing interests

The authors declare they have no competing interests, financial or other.

## Authors’ contributions

All authors listed have contributed sufficiently to the project to be included as authors, and all those who are qualified to be authors are listed in the author byline. NK conceived the original idea for the project. VJD worked on the design of the project with the input of NK. VJD carried out the data collection and analysis. VJD drafted the first version and subsequent revisions of the manuscript; NK reviewed and edited the various versions of the manuscript. Both authors approved the final draft.

## Pre-publication history

The pre-publication history for this paper can be accessed here:

http://www.biomedcentral.com/1472-6920/13/171/prepub
